# Preparation of Ag@3D‐TiO_2_ Scaffolds and Determination of its Antimicrobial Properties and Osteogenesis‐promoting Ability

**DOI:** 10.1111/os.14081

**Published:** 2024-05-05

**Authors:** Tiansheng Liu, Guijun Yang, Tong Li, Qi Wang, Houjiang Liu, Fang He

**Affiliations:** ^1^ Department of Orthopaedics Tianjin Hospital, Tianjin University Tianjin China; ^2^ School of Materials Science and Engineering and Tianjin Key Laboratory of Composite and Functional Materials, Tianjin University Tianjin China; ^3^ Department of Training and Sports Medicine Characteristic Medical Center of Chinese People's Armed Police Force Tianjin China

**Keywords:** Antimicrobial properties, Biocompatible, Osteogenesis‐promoting ability, Silver

## Abstract

**Objectives:**

The micro‐nano structure of 3D‐printed porous titanium (Ti) alloy with excellent performance in avoiding stress shielding and promoting bone tissue differentiation provides a new opportunity for the development of bone implants, but it necessitates higher requirements for bone tissue differentiation and the antibacterial properties of bone implants in clinical practice.

**Methods:**

This study investigated the preparation, antimicrobial properties, and osteogenesis‐promoting ability of the 3D printed porous Ti alloy anodic oxidized Ag‐carrying (Ag@3D‐TiO_2_) scaffolds. The 3D printed porous Ti alloy (3D‐Ti), anodized 3D printed porous Ti alloy (3D‐TiO_2_), and Ag@3D‐TiO_2_ scaffolds were synthesized using electron beam melting. The antimicrobial properties of the scaffolds were examined using antibacterial tests and their cytocompatibility was assessed using a cell proliferation assay and acridine orange/ethidium bromide (AO/EB) staining. In vitro cellular assays were used to investigate the effects of the scaffold microstructural features on cell activity, proliferation, and osteogenesis‐related genes and proteins. In vivo animal experiments were used to evaluate the anti‐inflammatory and osteogenesis‐promoting abilities of the scaffolds.

**Results:**

The Ag@3D‐TiO_2_ scaffolds exhibited sustained anti‐microbial activity over time, enhanced cell proliferation, facilitated osteogenic differentiation, and increased extracellular matrix mineralization. In addition, alkaline phosphatase (ALP), collagen type I (COL‐I), and osteocalcin (OCN)‐related genes and proteins were upregulated. In vivo animal implantation experiments, the anti‐inflammatory effect of the Ag@3D‐TiO_2_ scaffolds were observed using histology, and a large amount of fibrous connective tissue was present around it; the Ag@3D‐TiO_2_ scaffolds were more bio‐compatible with the surrounding tissues compared with 3D‐Ti and 3D‐TiO_2_; a large amount of uniformly distributed neoplastic bone tissue existed in their pores, and the chronic systemic toxicity test showed that the 3D‐Ti, 3D‐TiO_2_, and Ag@3D‐TiO_2_ scaffolds are biologically safe.

**Conclusion:**

The goal of this study was to create a scaffold that exhibits antimicrobial properties and can aid bone growth, making it highly suitable for use in bone tissue engineering.

## Graphical abstract

Anodized and silver‐loaded titanium alloy scaffolds were prepared to study the antimicrobial properties, biocompatibility and bone‐enhancing ability, and the Ag@3D‐TiO_2_ scaffolds were found to have good osseointegration ability.

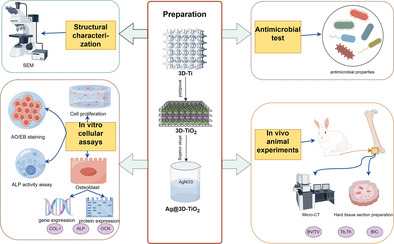

## Introduction

With the rapid development of social modernization and rapid entry into an aging society, the number of orthopedic patients with conditions such as osteoarthrosis, spinal diseases, osteoporosis, and trauma is rapidly increasing, followed by an explosive increase in the demand for orthopedic implants and implantable medical devices. By 2030, the total number of hip replacements is projected to increase by 174% compared with 2007, and the number of total knee replacements is projected to increase by 673% annually.^1^ As orthopedic surgery advances, the number of complications related to implants also increases. Prosthetic joint infection (PJI) is particularly serious because it often occurs after hip and knee arthroplasty and presents one of the most challenging obstacles in orthopedic clinical practice.^2^ Kurtz *et al*. found that there was a significant increase in the incidence of PJI after joint replacement from 2001 to 2019,^1^, ^2^, ^3^ which not only brings great pain to patients but also causes great burden to the social economy.^4^ Therefore, it is important to develop new implantable materials that effectively inhibit bacterial attachment and contribute to osteogenic differentiation.

Titanium (Ti) alloys have attracted significant interest in the field of orthopedic implants owing to their exceptional biodegradability, optimal density, and corrosion.^5^, ^6^ However, Ti alloys are biologically inert, with insufficient osteogenic capacity and high modulus of elasticity, which can lead to the occurrence of a “stress shielding” effect and inflammatory infections within the body over an extended period. This can ultimately hinder the formation and reconstruction of new bone and increase the risk of secondary surgery.^5^ Porous material have an elastic modulus matching with human bone tissue, which is an effective method to avoid “stress shielding;” however, the traditional preparation method has an uneven pore structure, limited adjustment range, and is prone to residual porogenic agents.^7^ In recent years, the development of 3D printing technology has provided a new research way to creating porous metals with controllable composition and structure. In the field of orthopedic implant design, the use of 3D printing technology has become a popular area of investigation for clinical applications.^8^, ^9^, ^10^ This technology allows for precise control of performance parameters of the scaffold, resulting in porous Ti alloy materials with varying levels of porosity and pore sizes,^11^, ^12^ which match the elastic modulus of human bone, whereas the pore structure facilitates cell adhesion within the scaffold. The majority of current research on 3D printed porous alloy focuses on the mechanical properties of various pore structures and their effects on bone tissue development. However, similar to ordinary Ti alloy implant materials, 3D‐printed Ti alloys without surface treatment have poor antibacterial properties and still have a high risk of postoperative infection.

The porous Ti alloy surface structure increases the area of bacterial attachment, leading to a higher risk of PJI in implants. According to previous studies, building specific nanostructure arrays on the implant surface can prevent the production of bacteria,^13^, ^14^ such as the porous Ti alloy surface is formed by secondary anodization to form an oxidized film that is biologically active and inhibits the production of bacteria to a certain extent.^15^, ^16^, ^17^ However, for orthopedic implant materials, it is important to exhibit strong antimicrobial properties, good biocompatibility, and promote osteogenic differentiation in addition to inhibiting bacteria. Therefore, the biomedical properties of Ti alloys can be improved by modifying the surface with coatings.^18^, ^19^ However, there is still scarce research on surface modification of 3D printed porous Ti alloys. Silver (Ag) has garnered widespread attention because of its ability to exhibit high antibacterial activity, extended antimicrobial duration, and its resistance to developing drug resistance, among other factors. Wang *et al*. discovered that Ag has a substantial impact on eliminating antibiotic‐resistant bacterial strains.^20^ Yuan *et al*. reported that the inclusion of an Ag coating significantly enhanced the antimicrobial properties against biocompatible *Escherichia coli* and *Staphylococcus aureus*.^21^ Zheng *et al*. discovered that Ag ions exhibited antimicrobial effects against primary cariogenic bacteria found in the oral cavity and significantly increased the osteoblast expression.^22^ Therefore, Ag coatings have the advantages of good biocompatibility, low bacterial infection rate, low biological toxicity, and low degradation rate in vivo, which can be beneficial for clinical applications. This supports the development of 3D printed porous Ti alloy micro‐nano structures with improved bone tissue differentiation and antibacterial characteristics.

**FIGURE 1:**
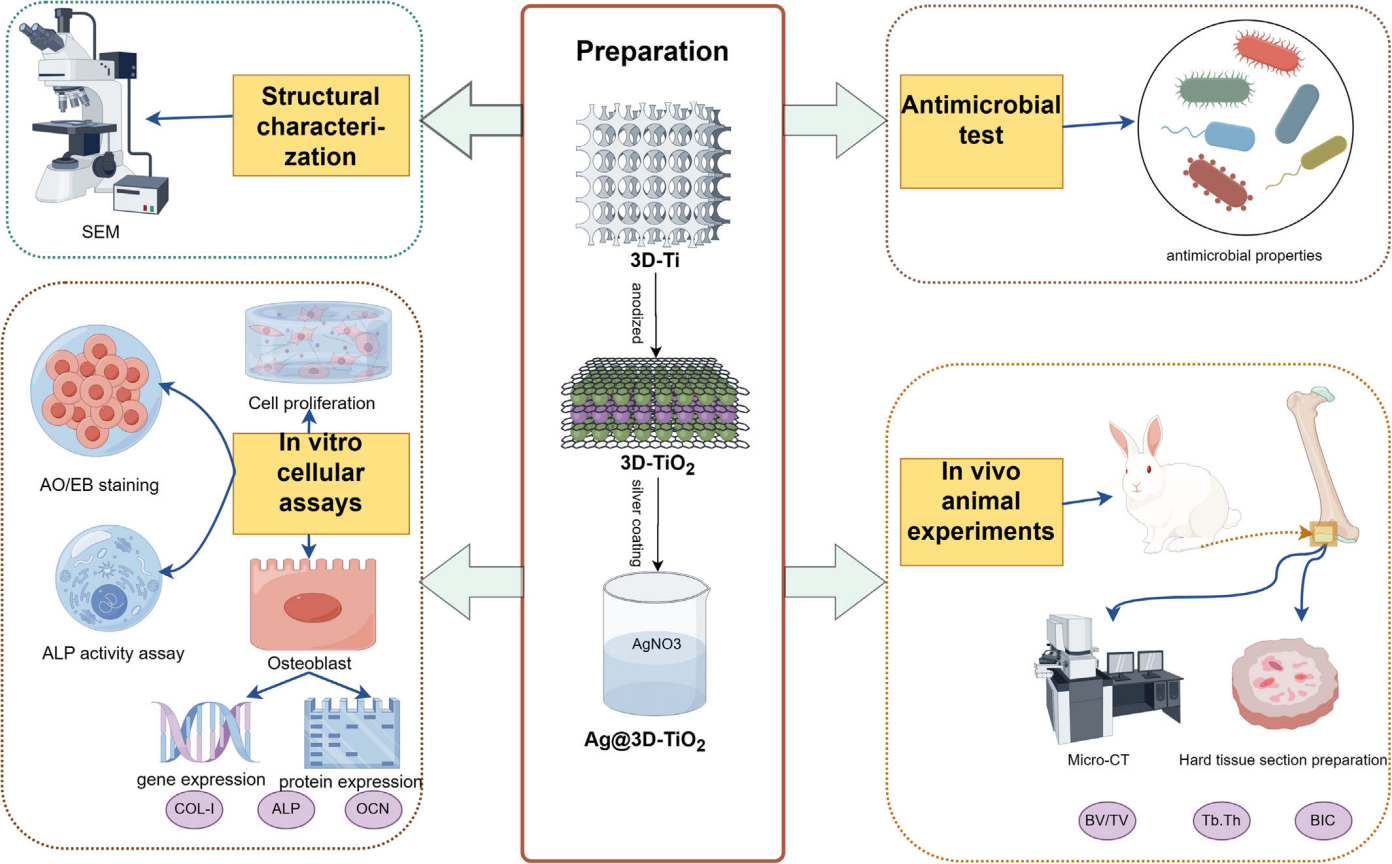
A diagram of the overall mechanism, By Figdraw.

Therefore, this study conducted anodizing on the 3D‐printed porous Ti alloy optimized by structure mechanical properties, constructed titanium dioxide nanostructures on its surface, and loaded Ag (Ag@3D‐TiO_2_) to significantly improve its biocompatibility and bone tissue differentiation ability, while also improving its antibacterial properties. The mechanism of antibacterial performance of the scaffold surface was investigated through cellular experiments to study the antibacterial effect of the scaffold surface against *S. aureus*. Proliferation, and osteogenesis‐related genes and proteins were explored, and finally, the scaffolds were subjected to in vivo animal experiments to study the osteogenesis‐promoting ability. The overall mechanism is illustrated in Figure 1.

## Materials and Methods

### Preparation of Scaffolds

In this experiment, we used a Ti‐6Al‐4V Ti alloy as a raw material, designed using the computer‐aided design of electron beam melting technology, and adjusted the technical parameters of 3D printing to prepare a Ti‐6Al‐4V porous Ti alloy material. According to previous studies, the porous Ti alloy with 60% porosity has good mechanical properties, the maximum compressive strength is about 112 MPa, and matches well with natural bone.^15^ Therefore, we chose a 3D printed porous Ti alloy scaffold with 60% porosity (filament diameter of 5.0, pore size of approximately 350 μm) for the subsequent modification study.

The 3D printed porous Ti alloy scaffold (3D‐Ti) was first cleaned with acetone for 15 min to remove organic impurities from the sample surface, isopropyl alcohol for 15 min to remove inorganic impurities, anhydrous methanol for 15 min to remove dust, and finally with deionized water for comprehensive cleaning.^23^ Subsequently, the dried specimens were acid washed for 10 s using a mixture of acids with a volume ratio of V(HF):V(HNO_3_):V(H_2_O) of 2:3:11.^24^ Following the acid washing step, the TiO_2_ nanolayers were prepared via electrochemical secondary anodization. In this process, the 3D printed porous Ti alloy served as the anode and a Pt sheet electrode was used as the cathode. The electrolyte was a fluorinated glycol solution with a V(deionized water):V(glycol) ratio of 3:97 containing 0.5% ammonium fluoride by mass. The process parameters, including DC power supply voltage and anodic oxidation time, were adjusted accordingly. After the first anodizing procedure, the samples were dried and ultrasonically washed with deionized water. This procedure was performed for 30 min. Subsequently, a second anodization step was performed using the same procedure. After the second anodization, the samples were annealed to remove residual fluorine and complete the crystalline phase transition of TiO_2_, Subsequently, they were kept at 450°C for 30 min at an average rate of 1°C min^−1^, and then cooled in the furnace to obtain anodized 3D printed porous Ti alloy scaffolds (3D‐TiO_2_).

**FIGURE 2:**
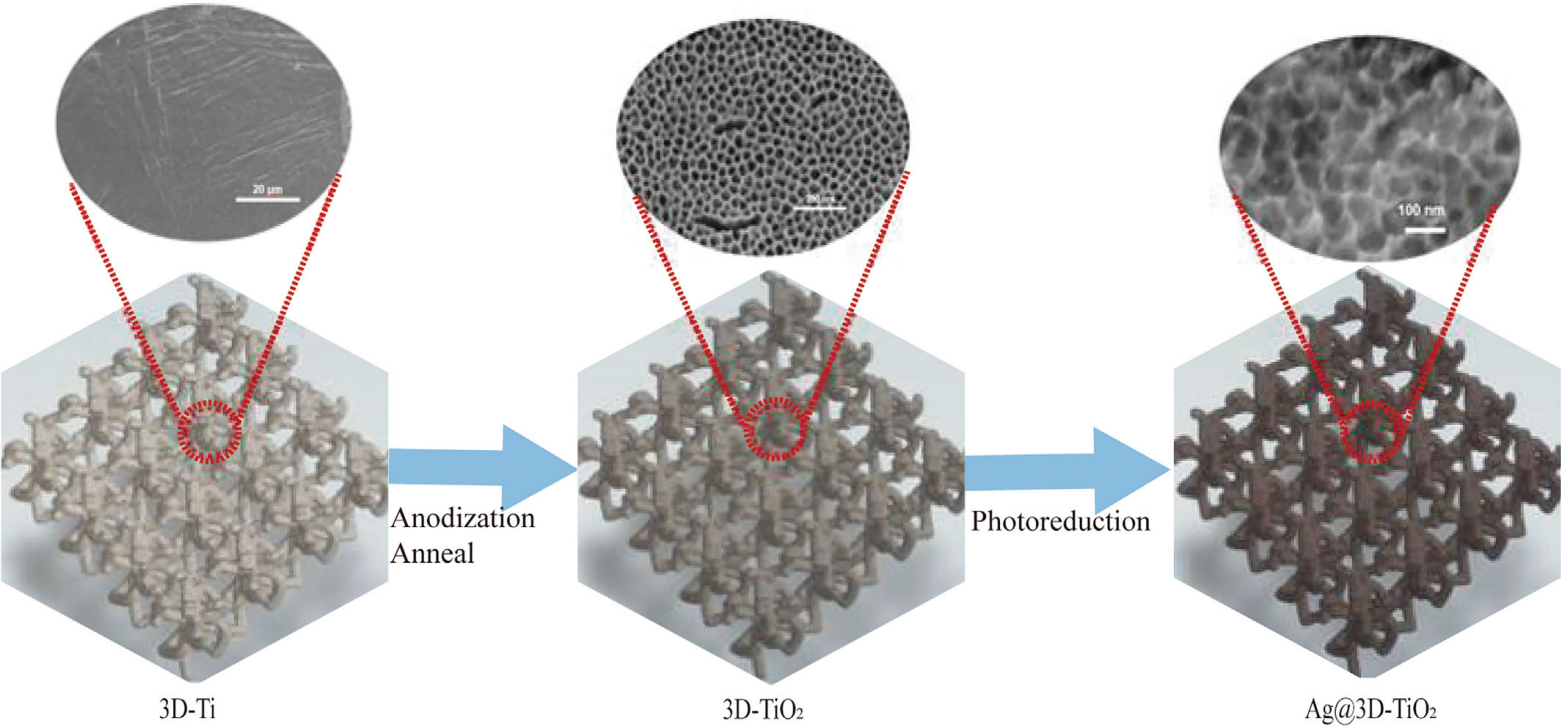
Schematic diagram of the preparation process.

For 30 min, the melted 3D‐TiO_2_ samples were submerged in a 0.1 M nitrate silver solution. They were then rinsed with deionized water, dried, and photographed under an intensity of 100 mW cm^−2^ (AM 1.5G) light for 2 h. The 3D printed porous Ti alloy anodic oxidized Ag‐carrying scaffolds loaded with monolithic Ag (Ag@3D‐TiO_2_) were obtained. The preparation process is illustrated in Figure 2.

The samples were divided into three groups, 3D‐Ti, 3D‐TiO_2_, and Ag@3D‐TiO_2_.

### Structural Characterization

The morphological characteristics, surface morphology, and coating dispersion of the scaffolds were examined by field‐emission scanning electron microscopy (FE‐SEM; S‐4800, Tokyo, Japan).

### Antimicrobial Test

For our antimicrobial studies, we selected the gram‐positive bacterium *S. aureus*,^15^ which was initially incubated in 5 mL of L.B. medium. Liquid medium containing 100 μL of the strain was dropped on a support and shock aerobically incubated overnight at 37°C, the supernatant was discarded, AO/EB (1:1 configuration) was added dropwise, and the antimicrobial effect was visualized using confocal microscopy after 10 min. The samples were exposed to a UV lamp, placed in a bacterial solution, and continuously shaken at 180 rpm at 37°C. Following incubation for 1, 3, 5, and 7 days, the samples were removed, and the concentration of the bacterial solution was measured at 600 nm using enzyme labels.

### In Vitro Cellular Assays

#### Cell Proliferation

MC3T3‐E1 cells were purchased from the National Collection of Authenticated Cell Cultures (Shanghai, China). Cells were cultured in α‐minimum essential medium (α‐MEM) supplemented with 10% FBS and 1% antibiotics (100 μg/mL streptomycin and 100 U/mL penicillin) at 37°C in a humidified atmosphere of 5% CO_2_.^25^ MC3T3‐E1 cells were divided into several groups: 3D‐Ti group, 3D‐TiO_2_ group, and Ag@3D‐TiO_2_ group. After treatment for 48 h, the cells were collected for further analysis. After 1, 3, and 5 days of incubation, 20 μL MTT (5 mg/mL) was added to each well. The samples were incubated with MTT in an incubator at 37°C for 4 h. 150 μL of dimethyl sulfoxide (DMSO) was added to each well. Absorbance was recorded with a detector on an enzyme labeler at 450 nm and cell proliferation was determined by absorbance reading.

#### AO/EB Staining

After 48 h of incubation, the cells were fixed for 20 min with 4% paraformaldehyde, rinsed three times in phosphate‐buffered saline (PBS) for 5 min each, stained according to the instructions of the AO/EB kit (Solarbio, Beijing, China),^26^ and dehydrated with gradient concentrations of 50%, 75%, and 100% ethanol. Confocal microscopy was used to observe apoptosis at 1, 3, and 5 days.

#### ALP Activity Assay

The ALP activities of 3D‐Ti, 3D‐TiO_2_, and Ag@3D‐TiO_2_ were determined using an ALP assay kit. MC3T3‐E1 cells were plated in 24‐well plates at a density of 1 × 10^5^ cells/mL and incubated with extract medium containing 50 mM ascorbic acid, 10 mM β‐glycerophosphate, and 100 nM dexamethasone for 7 days before being rinsed three times with PBS for 5 min each. The cells were stained using an Alkaline Phosphatase Assay Kit (Beyotime, Haimen, China), according to the manufacturer's stained with ALP. The instructions for ALP staining and ALP activity data were collected using a light microscope and analyzed semi‐quantitatively using the ImageJ software.

#### Alizarin Red Staining (ARS) Staining

ARS was used to identify mineralization of the extracellular matrix (ECM).^27^ MC3T3‐E1 cells were cultivated using an extract containing 50 mM ascorbic acid, 10 mM β‐glycerophosphate, and 100 nM dexamethasone after being seeded in 24‐well plates at a density of 1 × 10^5^ cells/mL. The cells were incubated for 21 days to induce osteogenesis. They were then fixed for 30 min with 4% paraformaldehyde solution and stained for an additional 30 min at ambient temperature with alizarin red solution. After rinsing three times with PBS for 5 min each to eliminate non‐specific staining, the stained cells were photographed using light microscopy, and ImageJ software was used to conduct semi‐quantitative studies of the mineralized ECM.

#### Osteogenesis‐related Gene Expression Assay‐real‐time Quantitative Polymerase Chain Reaction (RT‐qPCR)

Reverse transcription fluorescence RT‐qPCR was used to ascertain the effect of the 3D‐Ti, 3D‐TiO_2_, and Ag@3D‐TiO_2_ scaffolds on the expression of genes linked to osteogenesis.^28^ Sterilized 3D‐Ti, 3D‐TiO_2_, and Ag@3D‐TiO_2_ scaffolds were placed in a 6‐well plate. Following inoculation, the cells were grown for 1 week on the surface of each scaffold group. Then, in accordance with the manufacturer's recommendations, total RNA was extracted using the TRIzol reagent (Invitrogen, Carlsbad, CA, USA). After RNA was reverse‐transcribed into cDNA, primers created using a reverse transcription kit were used to amplify genes relevant to osteogenesis using cDNA as a template. Primers for ALP, COL‐I, and OCN were designed and analyzed using GAPDH as an internal reference^29^ (Table 1).

**TABLE 1:** Primer Sequences used in this study

Gene	Forward primer(5′‐3′)	Reverse(5′‐3′)
ALP	GTTGCCAAGCTGGGAAGAACAC	CCCACCCCGCTATTCCAAAC
COL‐I	GAAGGCTGGAGAGCGAG	GAAGGCTGGAGAGCGAG
OCN	GTGTGAGCTTAACCCTGC	ACAGGGAGGATCAAGTCC

Abbreviations: ALP, alkaline phosphatase; COL‐I, collagen type I; OCN, osteocalcin.

#### Protein Expression Levels

For total protein extraction, the samples were centrifuged at 1000 rpm for 3 min and rinsed three times with PBS, followed by the addition of 20 μL of PMSF lysate. When the cells were fully lysed, they were transferred into an EP tube and stored at −80°C until sufficient lysis of cells. The protein levels in the isolated samples were assessed using the BCA protein assay kit (LEAGENE, Beijing, China).

For western blot analysis of protein expression, 20 micrograms of protein were isolated using SDS‐PAGE and then electrotransferred onto PVDF membranes. The PVDF membranes were blocked using Tris‐buffered saline and TEMED solution containing 5% skimmed milk. ALP, COL‐I, and OCN from Santa Cruz Biotechnology, Texas, USA, were added to the membranes at a concentration of 1:1000, and the incubation period was 4°C. The membranes were then soaked in TBST three times for 5 min each. To find immunoreactive bands, samples were treated for 1 h with HRP‐conjugated secondary antibodies (1:5000; Santa Cruz, CA, USA). The blots were visualized using an ECL reagent (Boster, Wuhan, China).

### In Vivo Animal Experiments

#### Bone Implant Surgery

Eighteen New Zealand Large White rabbits (3–5 months old, weighing 2–2.5 kg) were randomly assigned into three groups, each consisting of six rabbits. The rabbits in each group were implanted with 3D‐Ti, 3D‐TiO_2_, and Ag@3D‐TiO_2_ scaffolds, respectively. The Ethics Committee of Tianjin Hospital, Tianjin, China approved the standard for experimental animal operations. The lateral route for surface implantation was used to access the knee's lateral femoral condyle. After prefilling the femoral marrow cavity with a 2 mm hand‐operated drill, and after drop of *S. aureus* was added, the material was implanted. Ultimately, the incision was carefully closed, layer by layer, and given the right care. All surgical protocols were performed under sterile conditions. Rabbits were anesthetized by injection of 10% chloral hydrate 8 weeks after implantation and femoral samples were collected for subsequent experiments.

#### Micro‐CT Imaging Analysis

The collected femurs were cleaned of the superficial fascia, flushed with saline, and photographed to observe the macroscopic condition of the defect. The specimens were kept in 10% paraformaldehyde and tested using micro‐CT scanning to obtain two‐dimensional images of the repaired bone defects. We collected data on the following parameters: percentage bone volume (BV/TV), bone canal thickness (Tb.Th), number of new bone tracks (Tb.N), and degree of bone separation (Tb.Sp).

#### Hard Tissue Section Preparation and VG Staining

The obtained femoral samples were dehydrated using anhydrous ethanol at gradients of 70%, 80%, and 100% before being fixed with 10% paraformaldehyde, followed by infiltration with 7200 VLC resin gel at 30%, 50%, 70%, and 100%. They were then embedded in 100% 7200 VLC resin gel and sectioned for subsequent staining using a hard‐tissue slicer.

Sections of these hard tissues were exposed to intense light with a Von Kossa silver nitrate solution for 30 min, washed with distilled water for 1 min, and then exposed to a sea wave solution for 2 min. Van Gieson staining was then applied, and the BV/TV was calculated using the Bioquant Osteo software (Bioquant Image Analysis Corporation, TN, USA). The stained sections were examined under a light microscope.

#### Safranin O/Fast Green Staining

The collected femur samples were fixed with 10% formalin, decalcified for paraffin sectioning, routinely deparaffinized to water, Weigert's stain was added for 5 min, acidic differentiation solution was added for 15 s, washed with distilled water for 10 min, then immersed within the solid green staining solution for 5 min, and quickly washed with a weak acid fallout for 10–15 s. Safranin O stain was added and immersed for 5 min, and then washed by 95% ethanol for 3 s, anhydrous ethanol for 3 s, anhydrous ethanol dehydration for 1 min dehydration, and finally sealed with optical resin.

#### Chronic Systemic Toxicity Tests

Eight weeks after surgery, the heart, liver, spleen, lung, and kidney tissues were removed, in addition to the femoral specimens. Soft tissues were preserved in 10% paraformaldehyde solution, dried, embedded in paraffin, sectioned for histological examination, and photographed using a light microscope (Nikon, Tokyo, Japan).

#### Statistical Analysis

Comparisons were performed using SPSS software (version 22.0; IBM, Armonk, NY, USA), and the differences between the groups were compared using either paired or independent sample *t*‐tests. All the obtained data are shown as mean ± standard deviation, with a statistically significant difference denoted by *p* < 0.05.

## Results

### Structural Characterization

**FIGURE 3:**
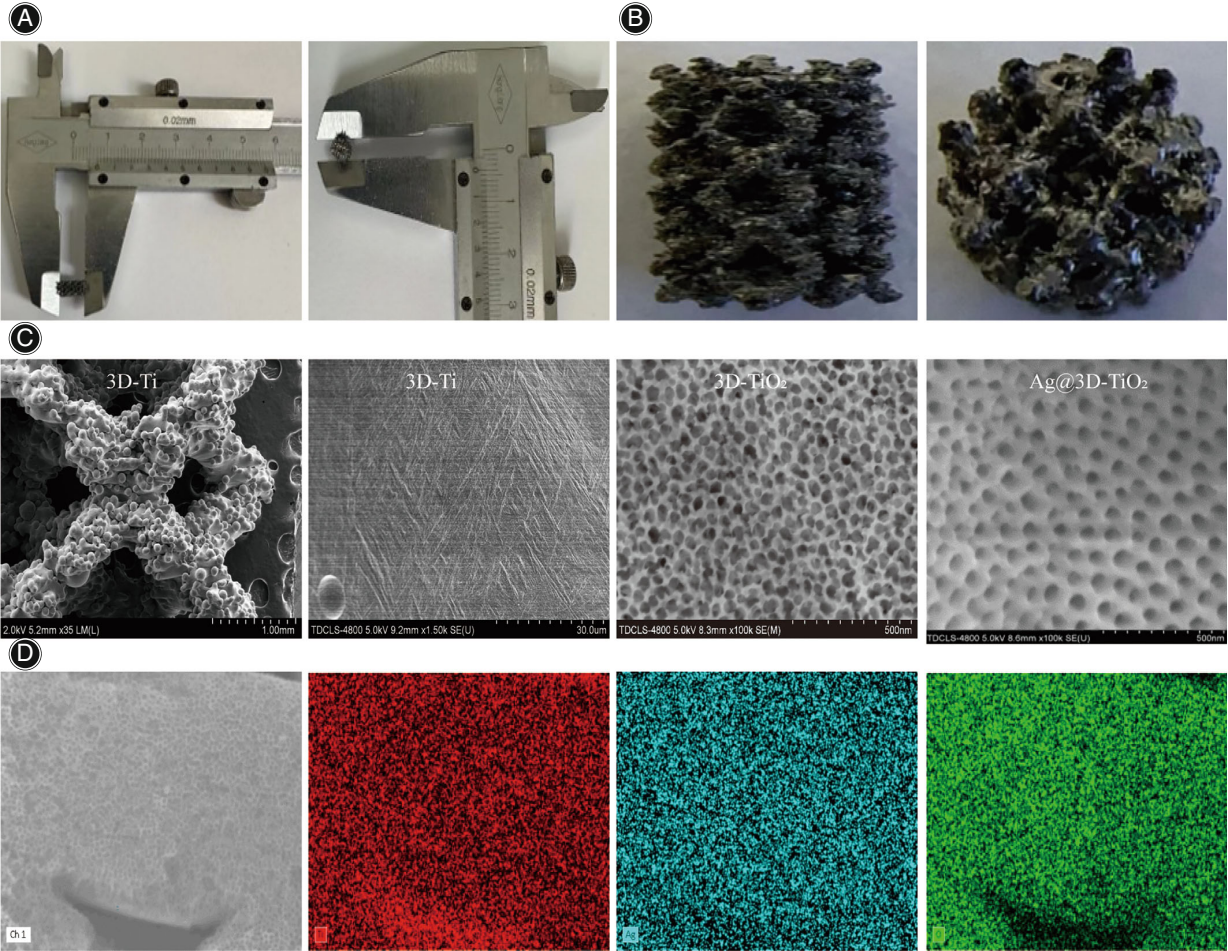
(A) Size of sample; (B) Surface porous structure of sample; (C) scanning electron microscopy images of surface t morphology of three scaffolds; (D) Distribution of Ag within TiO_2_ nanotubes.

The 3D‐Ti scaffolds contained samples that were 4 mm in diameter and 7 mm in height (Figure 3A), indicating rough porosity (Figure 3B). Figure 3C shows the SEM analysis of the three samples (3D‐Ti, 3D‐TiO_2_, and Ag@3D‐TiO_2_), depicting the surface morphologies. The 3D‐Ti surface morphology was rough, whereas the TiO_2_ nanolayers in the 3D‐TiO_2_ scaffolds covered the substrate surface uniformly without any detachment, indicating that the binding force between the TiO_2_ nanolayer and the matrix is strong and can grow relatively regular TiO_2_ nanotubes. The morphology of the TiO_2_ nanotubes in the Ag@3D‐TiO_2_ scaffold remained unchanged after the Ag loading. In addition, no significant Ag particles were observed on the surface of the scaffold, suggesting that the Ag particles were relatively small. SEM analysis shows that the distribution of Ag within the TiO_2_ nanotubes was relatively uniform compared with that of the Ag‐loaded coatings (Figure 3D).

### Antimicrobial Properties

**FIGURE 4:**
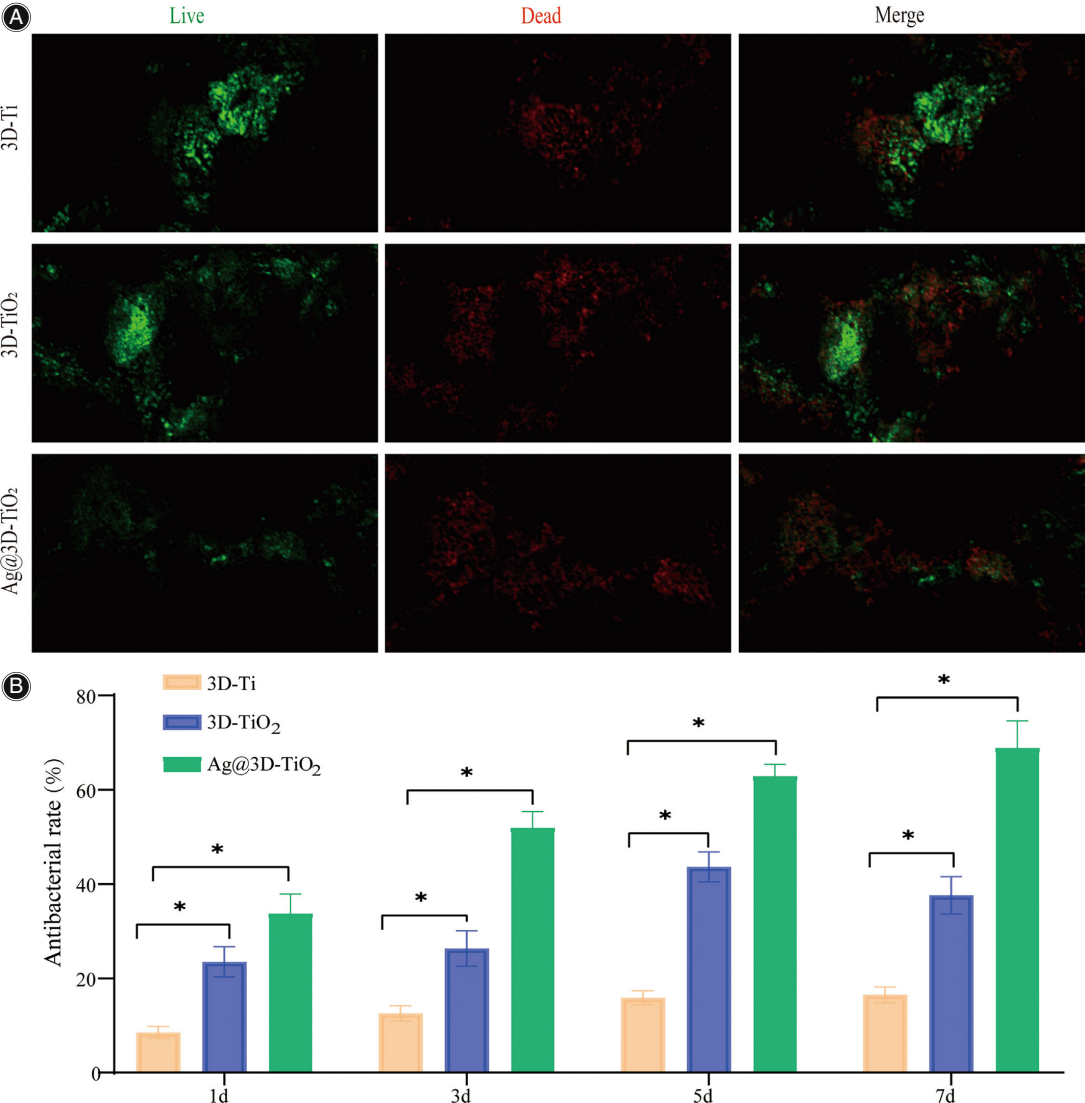
(A) Antimicrobial activity AO/EB staining of *Staphylococcus aureus* at 7 days; (B) Semi‐quantitative graphs of antimicrobial activity of *S. aureus* on 1, 3, 5, and 7 days; *p < 0.05. AO/EB, acridine orange/ethidium bromide.

The antibacterial effects of the 3D‐Ti, 3D‐TiO_2_, and Ag@3D‐TiO_2_ scaffolds were evaluated using antibacterial experiments. The results of AO/EB staining (Figure 4A) showed that numerous bacterial aggregates existed on 3D‐Ti after 7 days bacterial incubation period and a large green area in the merge plot. The bacteria in 3D‐TiO_2_ were slightly reduced compared with those in 3D‐Ti, and the proportion of the red‐green area in the merge plot was equivalent. The Ag@3D‐TiO_2_ scaffold showed a substantial reduction in bacterial count, with the majority of the area appearing red in the merged plot. This observation suggested that the Ag3D‐TiO_2_ scaffold exhibited the most effective antibacterial effect. From the semiquantitative results, after 1 day of culture, 3D‐TiO_2_ showed a significantly higher resistance bacterial rate against *S. aureus* compared with 3D‐Ti, approximately 2.75‐fold, that is, Ag@3D‐TiO_2_ was approximately 3.96‐fold higher, and the antibacterial rate of Ag@3D‐TiO_2_ was 1.44‐fold higher than that of 3D‐TiO_2_ scaffold. On day 7, compared with 3D‐Ti, the 3D‐TiO_2_ scaffold showed resistance against *S. aureus* approximately its 2.27‐fold and Ag@3D‐TiO_2_ scaffold approximately 4.17‐fold, and Ag@3D‐TiO_2_ scaffolds exhibited a 1.83‐fold increase in comparison with 3D‐TiO_2_ scaffolds, indicating a statistically significant *p* < 0.05 (Figure 4B). This result suggests that anodizing has the activity of inhibiting Staphylococcus aureus, and the use of surface silver coating effectively enhances its antibacterial effect.

### Evaluation of Biocompatibility and Osteogenic Capacity of Scaffolds

**FIGURE 5:**
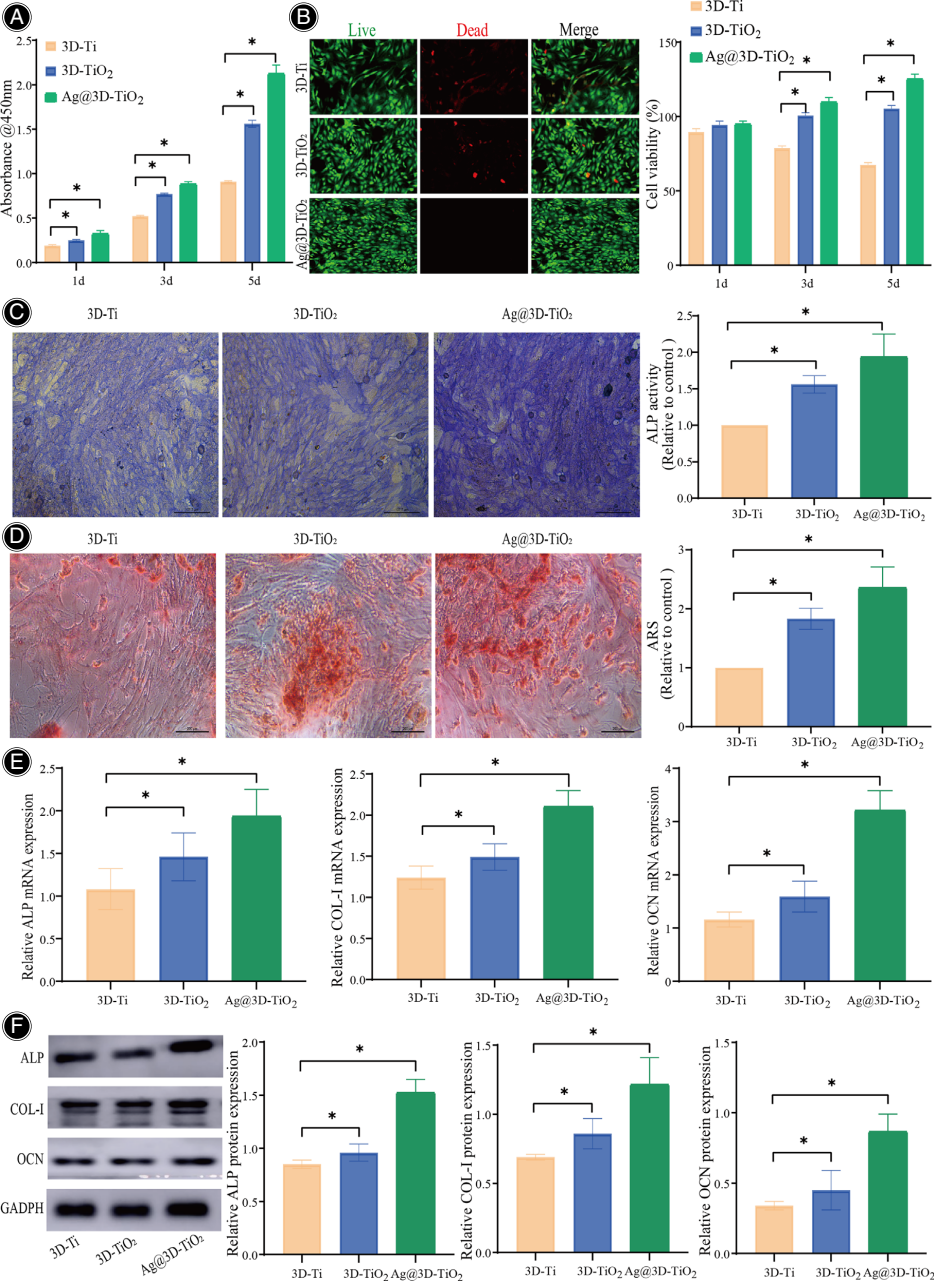
(A) Cell proliferation of 3D‐Ti, 3D‐TiO_2_, and Ag@3D‐TiO_2_ scaffolds for 1, 3, and 5 days. (B) The three scaffolds' cells were stained with AO/EB; (C) ALP staining of cells in the three scaffolds; (D) ARS staining of cells in the three scaffolds; (E) Expression of genes linked to OCN, COL‐I, and ALP in the three scaffolds; (F) Expression levels of the three scaffolds' ALP, COL‐I, and OCN proteins. *p < 0.05. AO/EB, acridine orange/ethidium bromide; ALP, alkaline phosphatase; ARS, Alizarin Red Staining; COL‐I, collagen type I; OCN, osteocalcin.

The biocompatibility of scaffolds serves as a vital criterion for evaluating orthopedic implants.^30^ Cell morphology after scaffold implantation significantly affects cell proliferation, differentiation, and ECM mineralization.^31^ The cell proliferation of the 3D‐Ti, 3D‐TiO_2_, and Ag@3D‐TiO_2_ scaffolds after co‐cultivation with cells for 1, 3, and 5 days is shown in Figure 5A. With the increase in culture time, the cell proliferation rates of all three scaffolds increased, compared with 3D‐Ti scaffolds, the cell proliferation rate of 3D‐TiO_2_ scaffolds increased, 1.56 ± 0.04 at the 5 days, the cell proliferation rate of Ag@3D‐TiO_2_ scaffolds was significantly increased by 2.13 ± 0.09. Based on these findings, the Ag@3D‐TiO_2_ scaffold exhibited a high rate of cell proliferation.

Apoptosis was detected through AO/EB staining.^32^ Compared with the 3D‐Ti scaffold, the 3D‐TiO_2_ scaffolds had fewer apoptotic cells and relatively more green fluorescence, whereas the Ag@3D‐TiO_2_ scaffolds had very few apoptotic cells, and a large cell population existed in the green coalescence, indicating that there were numerous cells and strong activity (Figure 5B). The variations in cell activity across the three scaffold groups after 1 day of cell activity calculations were not statistically significant. However, after 3 days, there was a significant increase in cell activity for the 3D‐TiO_2_ scaffolds (21.89% ± 1.36%) and 3D‐TiO_2_ scaffolds (31.46% ± 1.94%) compared with the 3D‐Ti scaffolds. The differences became even more pronounced after 5 days as the cell activity of the 3D‐TiO_2_ scaffolds increased by 37.91% ± 1.79% and that of the Ag@3D‐TiO_2_ scaffolds increased by 58.21% ± 2.03%, both of which were statistically significant (*p* < 0.05). This finding indicates that the Ag@3D‐TiO_2_ scaffolds can maintain the cell activity for a long time.

The early osteogenic differentiation abilities of the 3D‐Ti, 3D‐TiO_2_, and Ag@3D‐TiO_2_ scaffolds were assessed using alkaline phosphatase (ALP). ALP staining of the three scaffolds after 5 days of osteoblast‐induced differentiation^33^ is shown in Figure 5C. Moreover, the degree of ALP staining of the Ag@3D‐TiO_2_ scaffolds was significantly greater than those of the 3D‐Ti and 3D‐TiO_2_ scaffolds, and the distribution was uniform. Based on the semi‐quantitative graphs, there was a notable increase in ALP activity for 3D‐TiO_2_ scaffolds at a value of 1.56 ± 0.12 compared with the 3D‐Ti scaffolds. Additionally, the cellular activity of Ag@3D‐TiO_2_ scaffolds was significantly higher, measuring 1.94 ± 0.31, and the ALP activity of Ag@3D‐TiO_2_ scaffolds was statistically higher than that of 3D‐TiO_2_ scaffolds at 0.38 ± 0.18 (*p* < 0.05). This result suggests that the Ag@3D‐TiO_2_ scaffolds exhibited outstanding potential for differentiation into osteogenic tissues.

ARS staining was used to determine the extent of ECM mineralization during osteoblast differentiation.^34^ Figure 5D shows that the Ag@3D‐TiO_2_ scaffolds stained more intensely with ARS than the 3D‐TiO_2_ and 3D‐Ti scaffolds (orange), and were distributed in clumps with a significant mineralization level. According to the semi‐quantitative graphs, compared with 3D‐Ti scaffolds, 3D‐TiO_2_ scaffolds had a significantly higher level of ECM mineralization of 1.83 ± 0.18, Ag@3D‐TiO_2_ scaffolds had a significantly higher of 2.36 ± 0.35, and Ag@3D‐TiO_2_ scaffolds were statistically higher than those of 3D‐TiO_2_ scaffolds by 0.353 ± 0.17 (*p* < 0.05). This result implies that the scaffolds made with Ag@3D‐TiO_2_ exhibited a greater degree of ECM mineralization.

The effect of the scaffold on MC3T3‐E1 osteogenic differentiation‐related genes and protein expression levels was investigated.^35^ RT‐qPCR was used to evaluate the expression of the osteogenic differentiation‐related genes ALP, COL‐I, and OCN (Figure 5E). Compared with 3D‐Ti scaffolds, the expression of ALP, COL‐I, and OCN‐related genes was upregulated in 3D‐TiO_2_ scaffolds, corresponding to 1.46 ± 0.28, 1.49 ± 0.16, and 1.59 ± 0.29, respectively, with the change in ALP‐related genes being the highest, that is, 1.36‐fold of the 3D‐Ti scaffold value. ALP, COL‐I, and OCN‐related gene expression of the Ag@3D‐TiO_2_ scaffold were significantly increased to 1.94 ± 0.31, 2.11 ± 0.19, and 3.22 ± 0.36, respectively, with OCN‐related gene expression being the highest, that is, 2.78‐fold higher than the 3D‐Ti scaffold value. The expression of ALP, COL‐I, and OCN‐related genes was significantly higher in the Ag@3D‐TiO_2_ scaffold than in the 3D‐TiO_2_ scaffold, corresponding to fold increases of 1.33, 1.42, and 2.03, respectively.

Western blotting was performed to ascertain the amount of protein expression associated with osteogenic differentiation (Figure 5F). Compared with the 3D‐Ti scaffolds, the 3D‐TiO_2_ scaffolds exhibited higher levels of ALP, COL‐I, and OCN protein expression, with values of 0.96 ± 0.08, 0.86 ± 0.11, and 0.45 ± 0.14, respectively. The increase in OCN‐related protein expression was particularly notable, with a 1.32‐fold higher level than that on 3D‐Ti scaffolds. Furthermore, the Ag@3D‐TiO_2_ scaffolds showed significantly elevated expression of ALP, COL‐I, and OCN‐related proteins, with values of 1.53 ± 0.12, 1.22 ± 0.19, and 0.87 ± 0.12, respectively. The increase in OCN‐related protein expression was substantially pronounced, with a 2.56‐fold increase compared with that of the 3D‐Ti scaffolds. In addition, the expression of ALP, COL‐I, and OCN‐related proteins was significantly increased in the Ag@3D‐TiO_2_ scaffolds compared with that in the 3D‐TiO_2_ scaffolds, with corresponding fold changes of 1.29, 1.42, and 1.93, respectively. Differences were considered statistically significant (*p* < 0.05). The results showed that the gene and protein expression levels related to osteogenic differentiation of the Ag@3D‐TiO_2_ scaffolds were significantly higher than those of the 3D‐Ti and 3D‐TiO_2_ scaffolds, indicating a strong ability to induce osteogenesis.

### In Vivo Assessment of Bone Regeneration in Animal Experimental Scaffolds

#### Histological Observation

**FIGURE 6:**
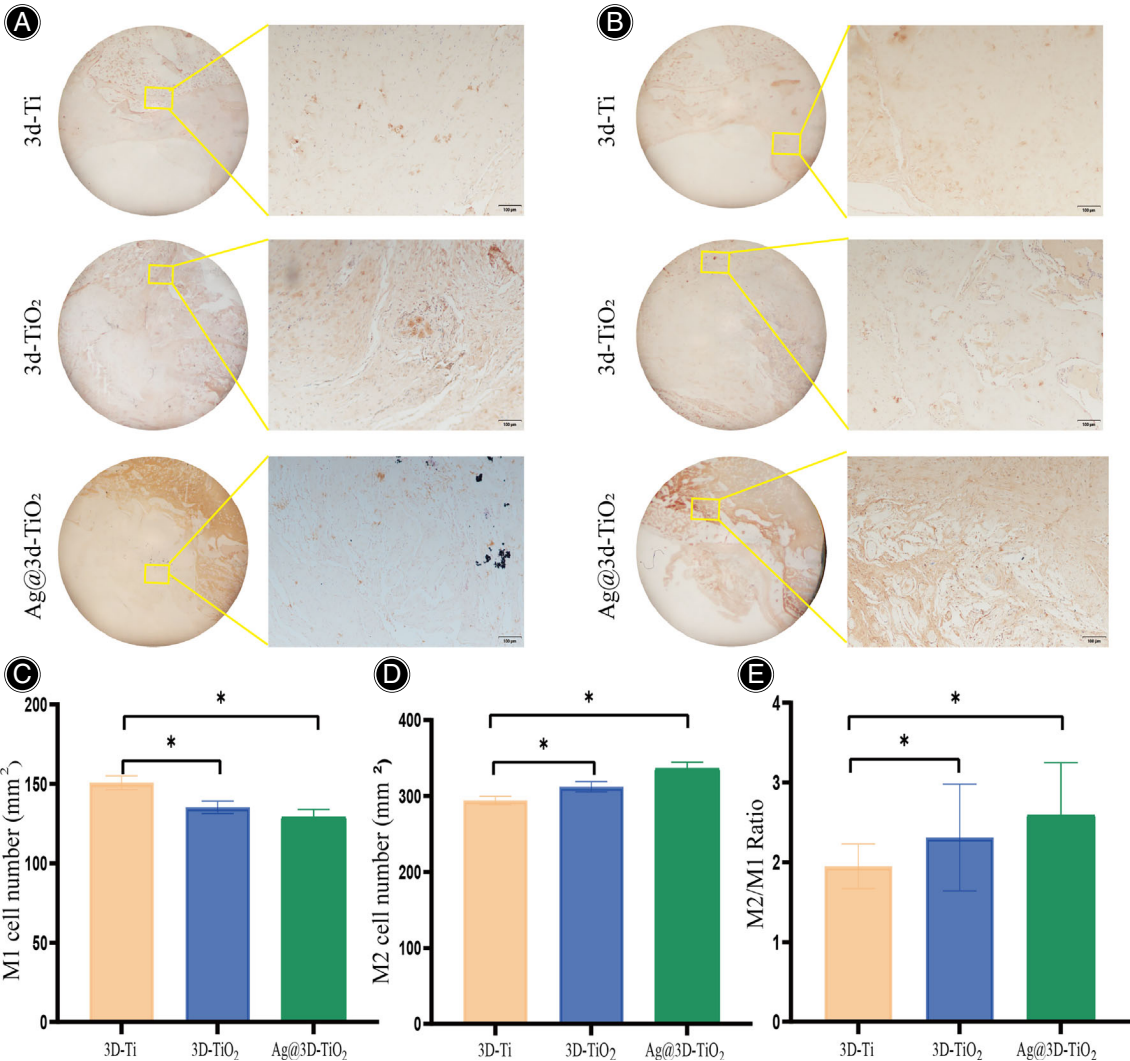
(A) Immunofluorescence staining of M1 macrophages; (B) Immunofluorescence staining of M2 macrophages; (C) Number of M1 macrophages; (D) Number of M2 macrophages; (E) Quantitative analysis of M2/M1 ratio *p < 0.05.

After implantation of the scaffold, immunohistological staining was used to observe the macrophage M1 and M2 polarization phenotypes of the scaffold on bone defects. It was found that the number of M1 macrophages in Ag@3D‐TiO_2_ scaffolds was significantly reduced compared with 3D‐Ti and 3D‐TiO_2_ scaffolds, whereas the number of M2 macrophages in Ag@3D‐TiO_2_ scaffolds was significantly increased compared with 3D‐Ti and 3D‐TiO_2_ scaffolds, while the M2/M1 ratio was significantly increased (Figure 6). This suggests that Ag@3D‐TiO_2_ scaffolds promoted macrophage polarization towards the M2 phenotype and reduced inflammation.

**FIGURE 7:**
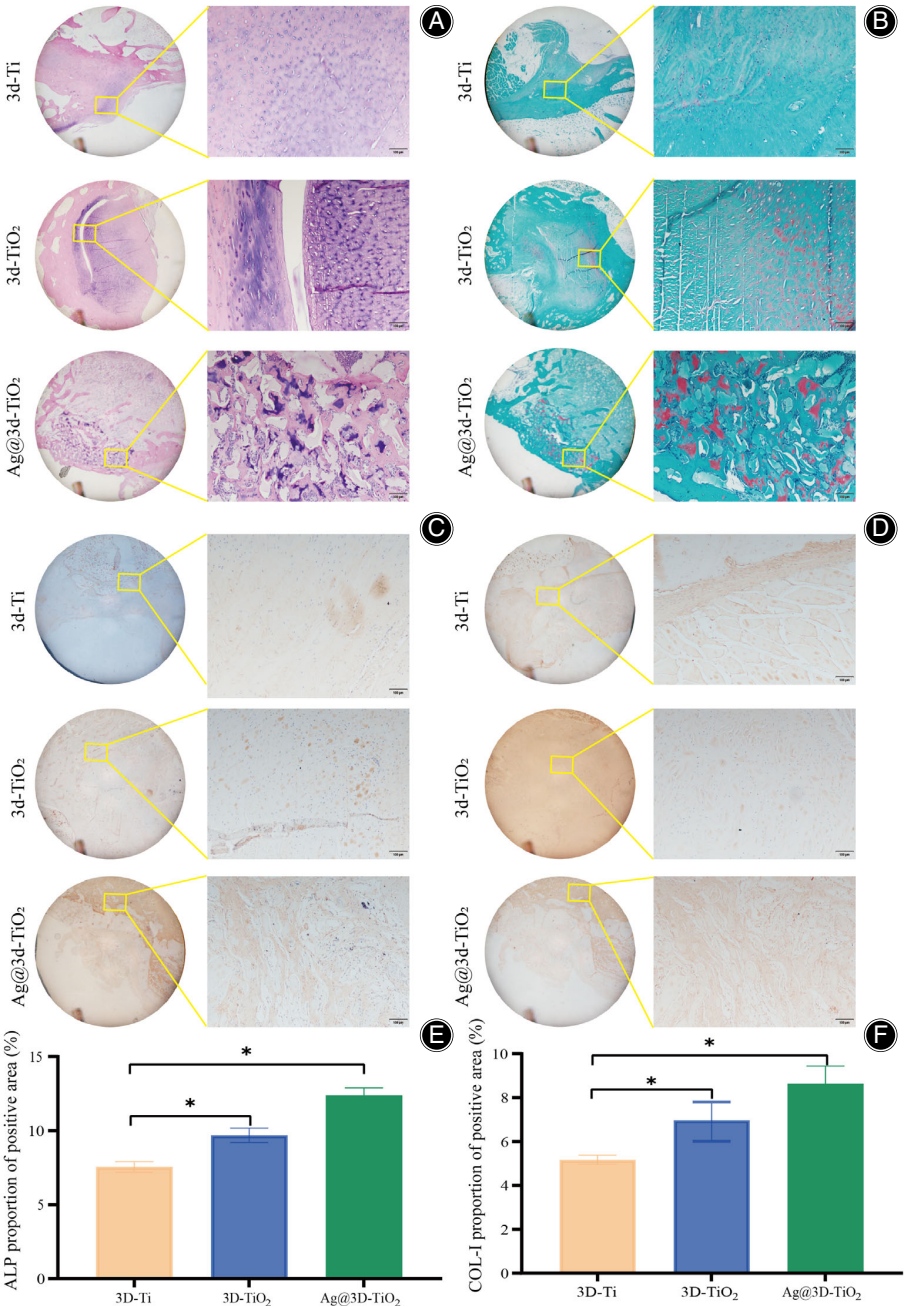
(A) HE immunohistochemical staining; (B) Safranin O/Fast green staining; (C) ALP immunohistochemical staining; (D) COL‐I immunohistochemical staining; (E) Proportion of positive areas in the images of ALP immunohistochemical staining; (F) Proportion of positive areas in the images of COL‐I immunohistochemical staining. *p < 0.05. ALP, alkaline phosphatase; COL‐I, collagen type I.

The neoplastic and fibrous tissues were stained with HE staining and Safranin O/Fast green staining, respectively, to observe the bone remodeling tissue after implantation. In H&E‐stained Figure 7A, there was only a small amount of fibrous tissue around the 3D‐Ti scaffold, a thin fibrous layer around the 3D‐TiO_2_ scaffold, and a uniform distribution of neoplastic bone tissue and fibrous connective tissue around the Ag@3D‐TiO_2_ scaffold. Safranin O/Fast green staining red showed cartilage tissue, very little red around the 3D‐Ti scaffolds, little linear red around the 3D‐TiO_2_ scaffolds, and a large amount of lumpy red around the Ag@3D‐TiO_2_ scaffolds (Figure 7B), suggesting that cartilage tissue has been generated from the Ag@3D‐TiO_2_ scaffolds.

The effect of scaffolds on bone remodeling in vivo was further assessed by immunohistochemical staining. ALP is one of the markers of early osteogenic differentiation. The ALP‐positive area of Ag@3D‐TiO_2_ scaffolds was the highest among all groups, and the ALP staining was also deeper, with a statistically significant difference of *p* < 0.05 (Figure 7C, E), which indicated that Ag@3D‐TiO_2_ scaffolds had a stronger bone regeneration capacity, which was consistent with the in vitro ALP staining results. Immunohistochemical staining of COL‐I, an osteogenesis‐related marker, showed that the expression of COL‐I in the Ag@3D‐TiO_2_ scaffold group was higher than that in the 3D‐Ti and 3D‐TiO_2_ scaffolds (Figure 7D, F).

#### Bone Regeneration of Scaffolds

**FIGURE 8:**
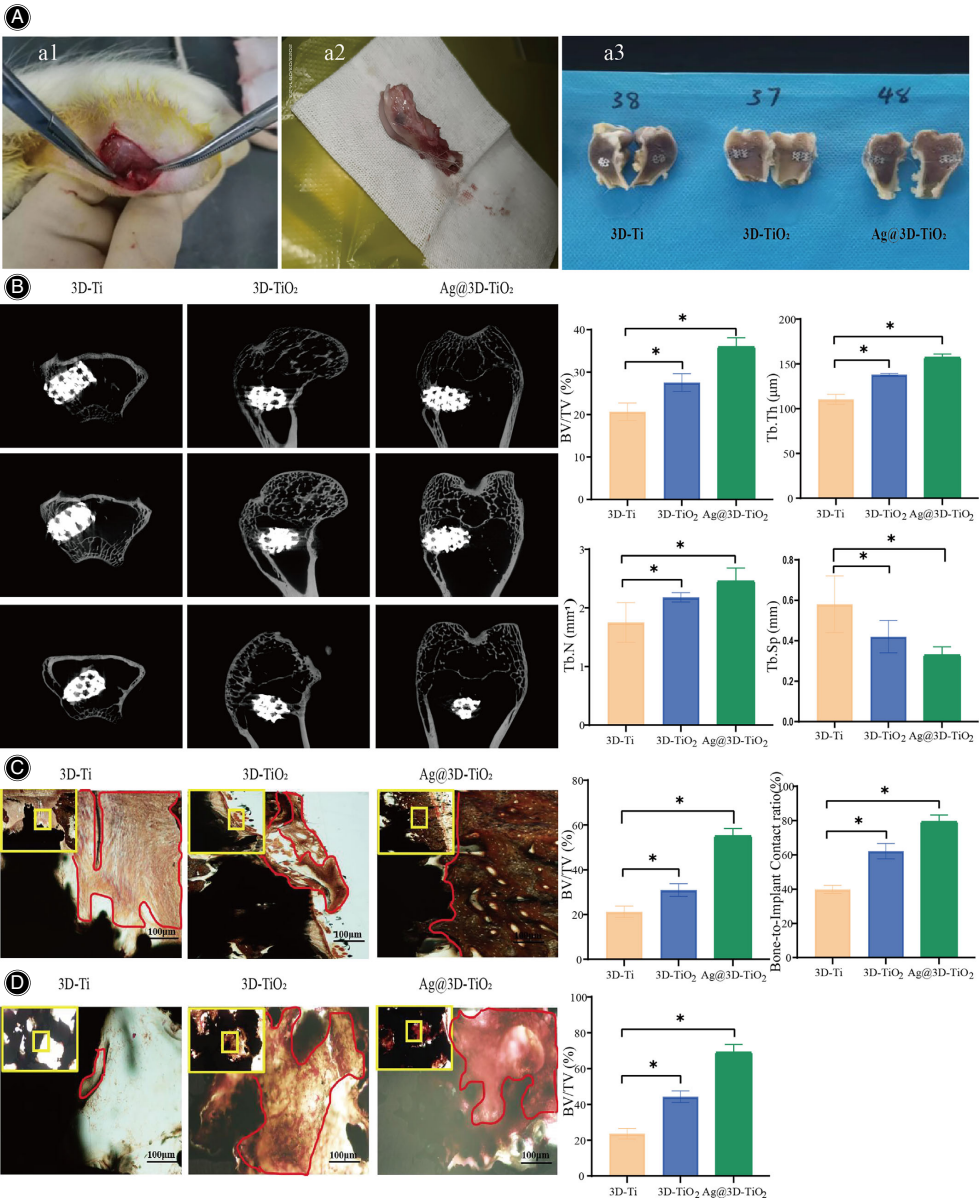
(A) a1‐Scaffold implantation was documented using macroscopic photographs. a2‐Specimens were sampled for examination. a3‐Femoral specimens were cut into two halves. (B) The three scaffolds were scanned using Micro‐CT. (C) The scaffold was observed to fuse with the surrounding bone tissue. (D) Bone growth within the scaffold pores was observed, with new bone tissue marked in red. *p < 0.05.

The 3D‐Ti, 3D‐TiO_2_, and Ag@3D‐TiO_2_ scaffolds were implanted into the lateral condyle of the femur of rabbits (Figure 8A‐a1). Eight weeks after implantation, a femoral specimen was obtained (Figure 8A‐a2), which was bisected and cut into two, and the three scaffolds were observed to grow new bone tissue (Figure 8A‐a3).

Micro‐CT of the cross‐sectional, sagittal, and coronal scan images of the three scaffolds showed the fusion of the implant and host bone interfaces (Figure 8B), with minimal new bone tissue present around the 3D‐Ti scaffolds. Some new bone tissue was generated around the 3D‐TiO_2_, and significantly more bone tissue was generated around the Ag@3D‐TiO_2_ scaffolds than around the 3D‐Ti and 3D‐TiO_2_ scaffolds, which formed mature mineralized bone tissues and fused well with the surrounding tissues. Based on semi‐quantitative analysis, the 3D‐TiO_2_ scaffolds exhibited increased BV/TV, Tb.Th, and Tb.N compared with those of the 3D‐Ti scaffolds, with corresponding fold changes of 1.33, 1.25, and 1.24, respectively. Additionally, the Ag@3D‐TiO_2_ scaffolds showed significantly higher BV/TV, Tb.Th, and Tb.N values than those of the 3D‐TiO_2_ scaffold, with fold changes of 1.74, 1.14, and 1.41, respectively. Moreover, Tb.Sp bone separation was reduced in the 3D‐TiO_2_ scaffolds compared to the 3D‐Ti scaffolds, whereas the Ag@3D‐TiO_2_ scaffolds demonstrated significantly lower Tb.Sp compared with the 3D‐TiO_2_ scaffolds. These findings indicate that the indices of bone tissue proportion and thickness around the Ag@3D‐TiO_2_ scaffolds were superior to those around the 3D‐Ti and 3D‐TiO_2_ scaffolds, whereas the number and thickness of new bone tissue increased, and the fusion between the bone and scaffolds increased, thereby significantly enhancing the capacity for bone regeneration in the Ag@3D‐TiO_2_ scaffolds.

The micro‐CT results were verified by histomorphometric measurements of VG‐stained images of hard tissue sections.^36^ Upon fusion of the scaffold with the surrounding bone tissue (Figure 8C), more bone tissue was covered around the Ag@3D‐TiO_2_ scaffolds with a deep red color and was tightly distributed. The bone volume and thickness on the surface of the scaffolds were significantly higher than those of the 3D‐Ti and 3D‐TiO_2_ scaffolds. According to the semi‐quantitative graphs, the 3D‐TiO_2_ scaffolds exhibited a higher BV/TV of (9.73 ± 2.69)% compared with the 3D‐Ti scaffolds. Additionally, the Ag@3D‐TiO_2_ scaffolds showed a significant increase in BV/TV of (24.22 ± 3.06)% compared with the 3D‐TiO_2_ scaffolds. The scaffold with the highest BIC value was Ag@3D‐TiO_2_, which was two times higher than that of the 3D‐Ti scaffold and 1.28 times higher than that of the 3D‐TiO_2_ scaffold, indicating a statistically significant difference (*p* < 0.05).

For bone growth within the pores of the scaffolds (Figure 8D), very few muscle fibers and collagen fibers were present at the edge of the pores of the 3D‐Ti scaffold, large muscle and collagen fibers were present in the pores of the 3D‐TiO_2_ scaffolds, which were unmineralized osteoids. The Ag@3D‐TiO_2_ scaffold had a deep red color in the pores, which was full of mature bone tissues tightly bonded to the scaffolds. From the semi‐quantitative graph, the BV/TV of the Ag@3D‐TiO_2_ scaffold was significantly higher than that of the 3D‐Ti and 3D‐TiO_2_ scaffolds, exceeding that of the 3D‐Ti scaffolds by 2.93‐fold and the 3D‐TiO_2_ scaffolds by 1.56‐fold, which was statistically significant (*p* < 0.05). The above results confirm that the Ag@3D‐TiO_2_ scaffolds exhibited high osseointegration and stability, and excellent biocompatibility, both on the surface of the scaffolds and within the pores, promoting in vivo bone tissue formation.

#### Chronic Systemic Toxicity Tests

**FIGURE 9:**
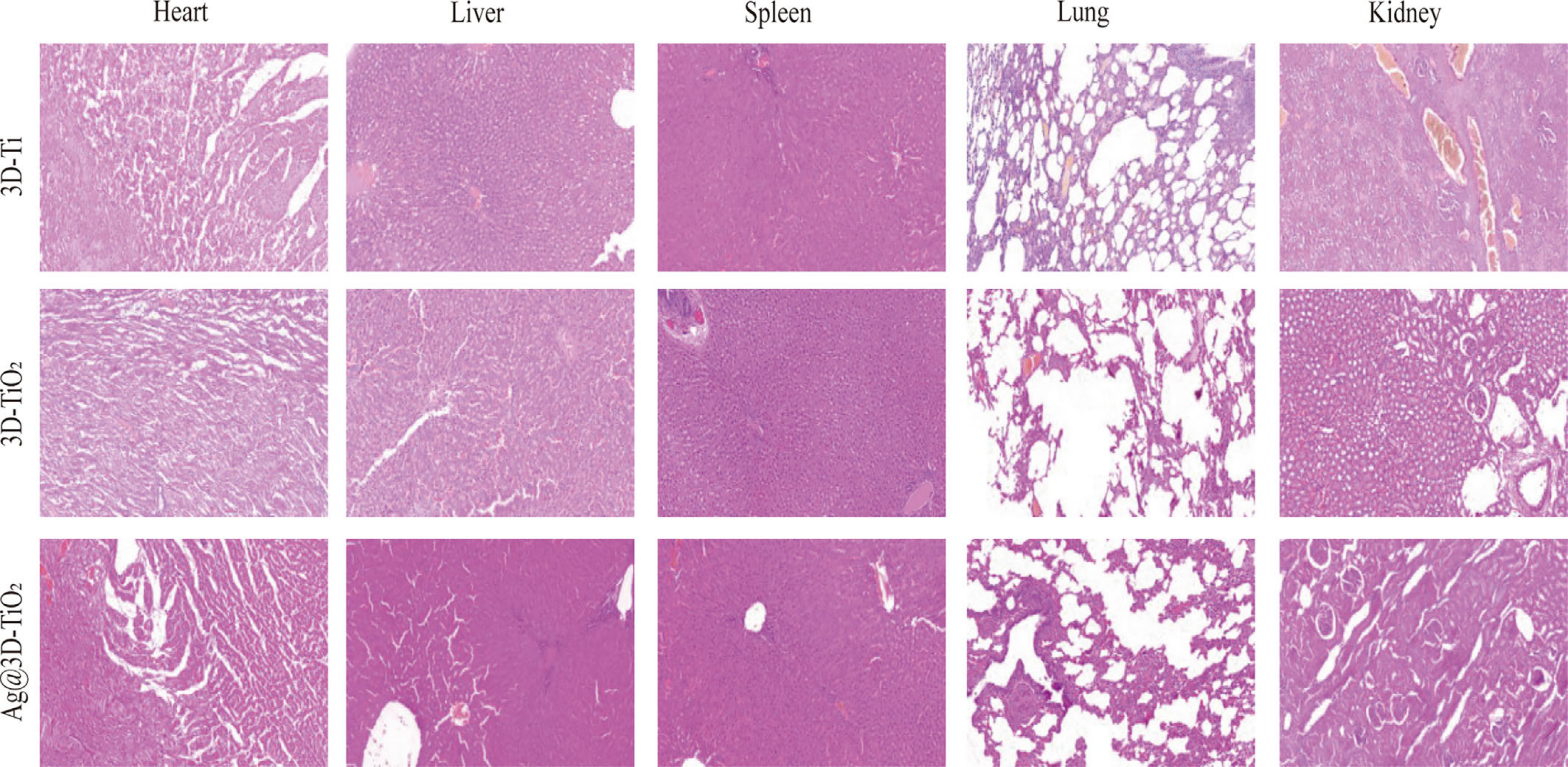
Heart, liver, spleen, lung, and kidney histological morphology following the implantation of the three scaffolds.

After implantation of the 3D‐Ti, 3D‐TiO_2_, and Ag@3D‐TiO_2_ scaffolds, biological safety issues should be considered, and H&E staining was applied to check the toxicity of the scaffolds.^37^ Eight weeks after implantation, the heart, liver, spleen, lung, and kidney tissues of the rabbits were analyzed. Figure 9 shows no evident pathological alterations in the three scaffolds, indicating that 3D‐Ti is biologically safe for implantation after anodic oxidation and silver‐loaded coating.

## Discussion

In this article, lightweight titanium alloy porous materials were prepared by 3D printing, anodized, titanium dioxide nanostructures were constructed on their surfaces and loaded with Ag to improve their antimicrobial properties, and the biocompatibility of the materials and the ability to differentiate bone tissues were significantly enhanced.

### Good Handling of Material Preparation

In order to counteract the “stress shielding” effect, 60% porous titanium alloy was selected for preparation, and the surfaces of the 3D‐Ti and 3D‐TiO_2_ scaffold structures were rough and porous, whereas the Ag@3D‐TiO_2_ scaffolds loaded with Ag did not change the morphology of the TiO_2_ nanotubes, and the distribution of Ag within the TiO_2_ nanotubes was more uniform, this indicates that the surface preparation treatment of the scaffolds is good.

### Improved Antimicrobial Properties of Scaffolds after Surface Silver Loading

The antimicrobial effect of 3D‐Ti on *S. aureus* was not ideal for 1 day of bacterial culture, while the antimicrobial rate of 3D‐TiO_2_ increased significantly compared with that of 3D‐Ti, and the antimicrobial rate of Ag@3D‐TiO_2_ increased significantly compared with that of 3D‐Ti; the antimicrobial rate of 3D‐TiO_2_ increased slower for 3 and 5 days of bacterial culture, but the antimicrobial rate of Ag@3D‐TiO_2_ continued to increase; the antimicrobial rate of bacteria cultured for 7 days, the antimicrobial rate of 3D‐TiO_2_ decreased significantly, while the antimicrobial rate of Ag@3D‐TiO_2_ increased steadily. This was also verified by the staining results, which showed that a large number of bacterial aggregates clearly existed on 3D‐Ti; the bacterial activity on the surface of 3D‐TiO_2_ was slightly reduced, and that of Ag@3D‐TiO_2_ was significantly reduced. It indicates that from the beginning of incubation, the anodic oxide film and the silver coating acted with obvious antibacterial performance, and with the increase of incubation time, the antimicrobial effect of 3D‐TiO_2_ tended to decrease, but the antimicrobial effect of Ag@3D‐TiO_2_ has been steadily increasing, confirming that the surface silver‐carrying coating significantly improves the long‐term antimicrobial performance.

### Improved Scaffolds Biocompatibility

In this article, in terms of cell proliferation, Ag@3D‐TiO_2_ has a higher cell proliferation rate compared with 3D‐Ti and 3D‐TiO_2_, which can maintain cell activity for a long time. Very few apoptotic cells and a large cell population existed in Ag@3D‐TiO_2_, indicating that the cells were numerous and active.

The ALP staining degree of Ag@3D‐TiO_2_ was significantly deeper than 3D‐Ti and 3D‐TiO_2_, and the ARS staining result was deeper than 3D‐Ti and 3D‐TiO_2_ with orange color, and the mineralization level was obvious, which indicated that the scaffold had a higher level of mineralization of extracellular matrix as well as the ability to contribute to the differentiation of bone. The expression of the genes related to the osteogenic differentiation of ALP, COL‐I, and OCN in the cells of Ag@3D‐TiO_2_ was significantly higher, and the expression of the proteins related to ALP, COL‐I, and OCN was higher, indicating that the scaffold had a stronger and more active cellular capacity. levels were elevated, indicating that the scaffolds had a strong osteogenic induction ability.

### Ability of Scaffolds to Promote Differentiation of Bone Tissue

In vitro animal experiments, the Ag@3D‐TiO_2_ scaffolds promoted macrophage polarization towards the M2 phenotype with strong anti‐inflammatory capacity, consistent with the results of in vitro bacterial experiments. Histological observations revealed the presence of more fibrous connective tissue, up‐regulated expression of osteogenesis‐related markers ALP and COL‐I around the Ag@3D‐TiO_2_ scaffolds than 3D‐Ti and 3D‐TiO_2_ scaffolds, which had generated cartilage tissue. A substantial quantity of newly formed bone tissue was present around the Ag@3D‐TiO_2_ scaffolds. The bone tissue was evenly and closely distributed, and the bone morphology parameters (BV/TV, Tb.Th, and Tb.N) were considerably higher in the Ag@3D‐TiO_2_ scaffolds than those of the 3D‐Ti and 3D‐TiO_2_ scaffolds. Additionally, Tb.Sp bone separation was significantly lower in the Ag@3D‐TiO_2_ scaffolds than in the 3D‐Ti and 3D‐TiO_2_ scaffolds. According to the staining results of the hard tissue sections, the pores of the Ag@3D‐TiO_2_ scaffolds were filled with mature bone tissue and tightly integrated with the scaffolds, indicating good biocompatibility.

### Strengths and Limitations

In this article, a 3D‐printed porous titanium alloy scaffold was prepared with an anodic oxide film and silver‐carrying coating on the surface and its antimicrobial properties were significantly improved, with high biocompatibility after implantation in animals, promotion of osteogenic differentiation ability, and high integration ability with surrounding bone tissues, which improves the success rate of bone implantation in the clinic.

This study has some notable limitations: (i) small sample size—the in vivo animal experiments were conducted with a small sample size, which may introduce bias in the data and limit the generalizability of the findings. A larger sample size would provide more robust results; (ii) lack of in‐depth scaffold design study—the suitability of the scaffold material design was not extensively studied in this research. Further experiments are needed to observe the implantation under various factors and address the challenges encountered in clinical applications; and (iii) incomplete assessment of scaffold biocompatibility—although the study mentioned good biocompatibility of the Ag@3D‐TiO_2_ scaffolds, the assessment was limited to the staining results of hard tissue sections. Further investigation should include more comprehensive biocompatibility assessments, such as cell viability and inflammatory response analysis. Overall, while the study provides valuable insights into the antimicrobial properties and osteogenesis‐promoting ability of Ag@3D‐TiO_2_ scaffolds, these limitations highlight areas for further research and improvement in the clinical application of these scaffolds.

### Prospects of Clinical Application

3D‐printed porous titanium alloy scaffolds with anodic oxide film and silver‐carrying coating on the surface will become a precise medical technology widely used in the clinical field of bone repair. It can reduce the risk of implant infection, improve biocompatibility, shorten the time of implant osseointegration, increase the success rate of implantation, and reduce the medical burden of patients in clinical applications. Although Ag@3D‐TiO_2_ scaffolds show great potential in bone bioscaffolding, there are still some shortcomings that affect their clinical application. First, the design of scaffold materials has not been studied in depth for suitability, which is not universal in the field of bone repair, and more experiments are needed to observe the implantation situation under the conditions of various factors in order to cope with the various problems encountered in clinical application. Second, degradation experiments are lacking, understanding the degradation rate of the scaffold is important for its long‐term performance and compatibility with bone ingrowth. Future studies should investigate the degradation properties of the scaffold.

## Conclusions

With the progress of science and technology and the deepening of material research and development, the clinical requirements for orthopedic implants are getting higher and higher. In this project, a new type of biomaterial Ag@3D‐TiO_2_ scaffold is prepared by 3D printing technology, which is a lightweight titanium alloy porous material that conforms to the mechanical properties of human bone and avoids “stress shielding.” The anodic oxide film and silver‐carrying coating on the surface can improve the antimicrobial performance and bone‐enhancing ability, which is expected to have good prospects for solving the therapeutic difficulties of bone defects in the clinic.

In conclusion, digital orthopedic tissue engineering scaffolds are in the stage of rapid development, Ag@3D‐TiO_2_ scaffolds have a broad space for development in orthopedic clinical applications due to their cost‐effective and biocompatible applications, and it is believed that, through continuous research and innovation, subsequent research and development will be carried out to develop tissue engineering scaffolds for bone restoration with higher osteogenic properties, which will become an important driving force for the development of orthopedic clinics.

## Conflict of Interest Statement

The author(s) declared no potential conflicts of interest with respect to the research, authorship, and/or publication of this article.

## Author Contributions

Tiansheng Liu, Guijun Yang, and Tong Li contributed to the conception and design of the study. Qi Wang, Houjiang Liu, and Fang He contributed to data acquisition. Tiansheng Liu, Guijun Yang, Tong Li contributed to the execution of the experiments. Tiansheng Liu, Qi Wang, and Houjiang Liu analyzed and interpreted the data and drafted the manuscript. All the authors approved the final version of the manuscript to be published. All authors listed meet the authorship criteria according to the latest guidelines of the International Committee of Medical Journal Editors. All authors are in agreement with the manuscript.

## Ethics Statement

The Ethics Committee of Tianjin Hospital, Tianjin, China (2020 Medical Ethics Review 065) approved the standard for experimental animal operations.
